# Deep learned triple-tracer multiplexed PET myocardial image separation

**DOI:** 10.3389/fnume.2024.1379647

**Published:** 2024-04-11

**Authors:** Bolin Pan, Paul K. Marsden, Andrew J. Reader

**Affiliations:** School of Biomedical Engineering and Imaging Sciences, King’s College London, London, United Kingdom

**Keywords:** multiplexed positron emission tomography, image separation, deep learning, compartmental modeling, data-driven learning

## Abstract

**Introduction:**

In multiplexed positron emission tomography (mPET) imaging, physiological and pathological information from different radiotracers can be observed simultaneously in a single dynamic PET scan. The separation of mPET signals within a single PET scan is challenging due to the fact that the PET scanner measures the sum of the PET signals of all the tracers. The conventional multi-tracer compartment modeling (MTCM) method requires staggered injections and assumes that the arterial input functions (AIFs) of each tracer are known.

**Methods:**

In this work, we propose a deep learning-based method to separate triple-tracer PET images without explicitly knowing the AIFs. A dynamic triple-tracer noisy MLEM reconstruction was used as the network input, and dynamic single-tracer noisy MLEM reconstructions were used as training labels.

**Results:**

A simulation study was performed to evaluate the performance of the proposed framework on triple-tracer ([F18]FDG+Rb82+[Tc99m]sestamibi) PET myocardial imaging. The results show that the proposed methodology substantially reduced the noise level compared to the results obtained from single-tracer imaging. Additionally, it achieved lower bias and standard deviation in the separated single-tracer images compared to the MTCM-based method at both the voxel and region of interest (ROI) levels.

**Discussion:**

As compared to MTCM separation, the proposed method uses spatiotemporal information for separation, which improves the separation performance at both the voxel and ROI levels. The simulation study also demonstrates the feasibility and potential of the proposed DL-based method for the application to pre-clinical and clinical studies.

## 1 Introduction

Positron emission tomography (PET) is clinically recognized as a powerful modality to visualise and investigate functional activity in the brain (1), heart (2), and whole body (3). In cardiology, dynamic PET imaging has been widely used to measure myocardial perfusion and to connect cardiac efficiency with the metabolism of myocardial substrates (4). PET has been largely conducted using only one radiotracer per imaging session, meaning that images of different processes can only be acquired separately through multiple scans. To obtain information on, for example, glucose metabolism with [F18]FDG (5), myocardial perfusion and potentially also the ${\mathrm{Na}}^{+}$/$K^{+}$ pump with Rb82 (6), and to measure the mitochondrial membrane potentials with [Tc99m]sestamibi (7), three cardiac PET scans may be conducted separately to allow for the decay of one of the tracers. This prolongs the scanning time for the patient and therefore increases the radiation exposure due to multiple CT scans in PET-CT.

Multiplexed PET (mPET) offers the opportunity of observing multiple targets of interest with different tracers simultaneously in a single scan, providing more relevant or complementary information for clinical decision making, reducing the total examination time, and allowing perfect co-registration of the images for each tracer. In mPET imaging, multiple tracers are injected sequentially with an offset of several minutes in between the administrations, followed by a dynamic PET scan. The dynamic/static imaging measurements of each individual tracer are then separated and recovered from the obtained dynamic mPET images.

The main challenge in mPET imaging is that each tracer produces to indistinguishable 511 keV photon pairs, and thus no unique energy information to differentiate the source of each photon pair, meaning that the PET scanner measures the sum of the PET signals of all tracers (8). Several methods with dynamic PET scans and staggered injection protocols have been proposed for mPET signal separation based on differences in biodistribution kinetics and radioactive decay (9–11). Another widely studied method for mPET separation is based on multi-tracer compartment modeling (MTCM). Koeppe et al. introduced a dual-tracer compartment model to estimate the kinetic parameters of two C11-labeled tracers (12, 13). This method was further investigated with different dual-tracer combinations using simulation data (14–16), large animal data (17), and tumour imaging data (18). Black et al. subsequently explored the feasibility of using this method for the triple-tracer time-activity curve (TAC) separation of [F18]FDG, [Cu62]ATSM, and [Cu62]PTSM (19). However, the MTCM method is susceptible to noise and prone to falling into local minima, even when the noise level is low, because of the non-linearity of the fitting problem. To improve the separation performance of the MTCM method, Zhang et al. reformulated the conventional MTCM using fewer parameters by separating the linear part from the nonlinear part (20). Cheng et al. further incorporated the dual-tracer separation using the reformulated model into the image reconstruction process to reduce the influence of noise (21). However, each of the aforementioned methods assumes that the arterial input function (AIF) of each tracer is known, which limits their practical viability. Verhaeghe and Reader (22) proposed using a set of basic exponential decay functions convolved with the estimated tracer-specific generating functions to fit the dual-tracer TACs for separating [F18]FDG and multiple [O15]HO2 signals. Although this method does not require the AIF of each tracer, the solution to the fitting problem is non-unique because of the alternative estimation of the generating functions and the decay coefficients. In addition, principal component analysis (PCA) (14), generalised factor analysis (23), reference region models (24), basis pursuit (25), and spectral analysis with image-derived input functions (26) have been studied for mPET separation. The mPET signals can also be separated by assuming an additional high-energy γ photon emitted with positrons of one of the two tracers, therefore discriminating the different isotopes (27–30). However, these methods are only valid for some tracer combinations, i.e., a pure positron-emitting isotope and a positron-γ emitting isotope, limiting the selection of tracers (30).

In recent years, deep learning (DL) has received much attention in the area of mPET imaging. In comparison to the MTCM method, the supervised DL-based methods (i) separate the mPET signals without explicitly knowing the AIF of each tracer; (ii) have the ability to separate mPET signals using staggered or even simultaneous injection protocols; and (iii) sufficiently reduce the influence of noise in the separation process. DL-based methods for mPET imaging mainly fall into one of two categories: (i) learned post-separation of an mPET reconstruction, such as filtered back projection (FBP) (31, 32), maximum likelihood expectation maximisation (MLEM) (32–35), and alternating direction method of multipliers (ADMM) (32, 34, 36, 37), (ii) direct-learned mPET image separation from sinogram (38, 39). The direct-learned method has also been extended to the separation of simultaneous triple-tracers ([C11]FMZ+[C11]MET+[F18]FDG) PET imaging based on simulated data (40). Wan et al. proposed an unsupervised DL-based method for joint mPET image separation and segmentation (41). Apart from the use of DL techniques, a machine learning method based on a recurrent extreme gradient boosting algorithm has also been shown to outperform the MTCM method for dual-tracer TAC separation for a region of interest (ROI) (42).

In this work, we characterise the feasibility of separating dynamic triple-tracer myocardial PET images in the learned post-separation framework, which has not thus far been reported elsewhere in the literature. In particular, we propose a customised convolutional encoder-decoder (CED) to separate triple-tracer ([F18]FDG+Rb82+[Tc99m]sestamibi) activity images of the myocardium into activity images of each tracer and compare its separation performance with dual-tracer ([F18]FDG+Rb82) separation and MTCM-based separation in a simulation study.

## 2 Methods

### 2.1 Model of mPET imaging

A dynamic mPET scan records the spatiotemporal distribution of a mixed uptake of N tracers within a living organism. The multi-tracer activity concentrations (i.e., multi-tracer TAC^1^) of an image voxel (or in a ROI) at time t can be modeled as the linear superposition of the pharmacokinetic model of each tracer (16, 17, 19)

(1) $$\begin{array}{rl} & C^{\text{Multi}}(t;\kappa )=V_{B}S(t)\\ & +(1-V_{B})\sum_{n=1}^{N}\left[A^{\left(n\right)}(t)⊗R^{\left(n\right)}(t;\kappa^{\left(n\right)})\right]e^{-\lambda^{\left(n\right)}t}, \end{array}$$

where κ is a vector that contains all tracer kinetic parameters, $R^{\left(n\right)}(t)$ is the impulse response function of the nth tracer, $\lambda^{\left(n\right)}$ denotes the rate of radioactive decay, $A^{\left(n\right)}(t)$ is the tracer concentration in plasma (i.e., AIF), S(t) is the total activity concentration in whole blood, $V_{B}\in (0,1]$ is the fractional volume of blood in the tissue, and ⊗ denotes the convolution operator. The mPET image intensity at voxel j in time frame k is then given by Equation 2:

(2) $$x_{k}^{\mathrm{Multi}}(\kappa_{j})=\int_{t_{k,s}}^{t_{k,e}}C^{\mathrm{Multi}}(\tau ;\kappa_{j})d\tau ,$$

where $t_{k,s}$ and $t_{k,e}$ represent the start and end points of frame k. The expectation of the projection mPET data ${\bar{y}}_{k}(\kappa )$ in time frame k with respect to the dynamic mPET image $x_{k}(\kappa )$ can be expressed by Equation 3:

(3) $${\bar{y}}_{k}^{\mathrm{Multi}}(\kappa )=Px_{k}^{\mathrm{Multi}}(\kappa )+r_{k},$$

where the (i,j)th element of the system matrix $P\in R^{I\times J}$ is the probability of detecting an event originating in voxel j by detector pair i, I and J are the total number of detector pairs and image voxels, respectively, and $r_{k}$ is the expectation of scattered and random events in the kth frame.

### 2.2 Deep learned triple-tracer PET image separation

**Figure 1:**
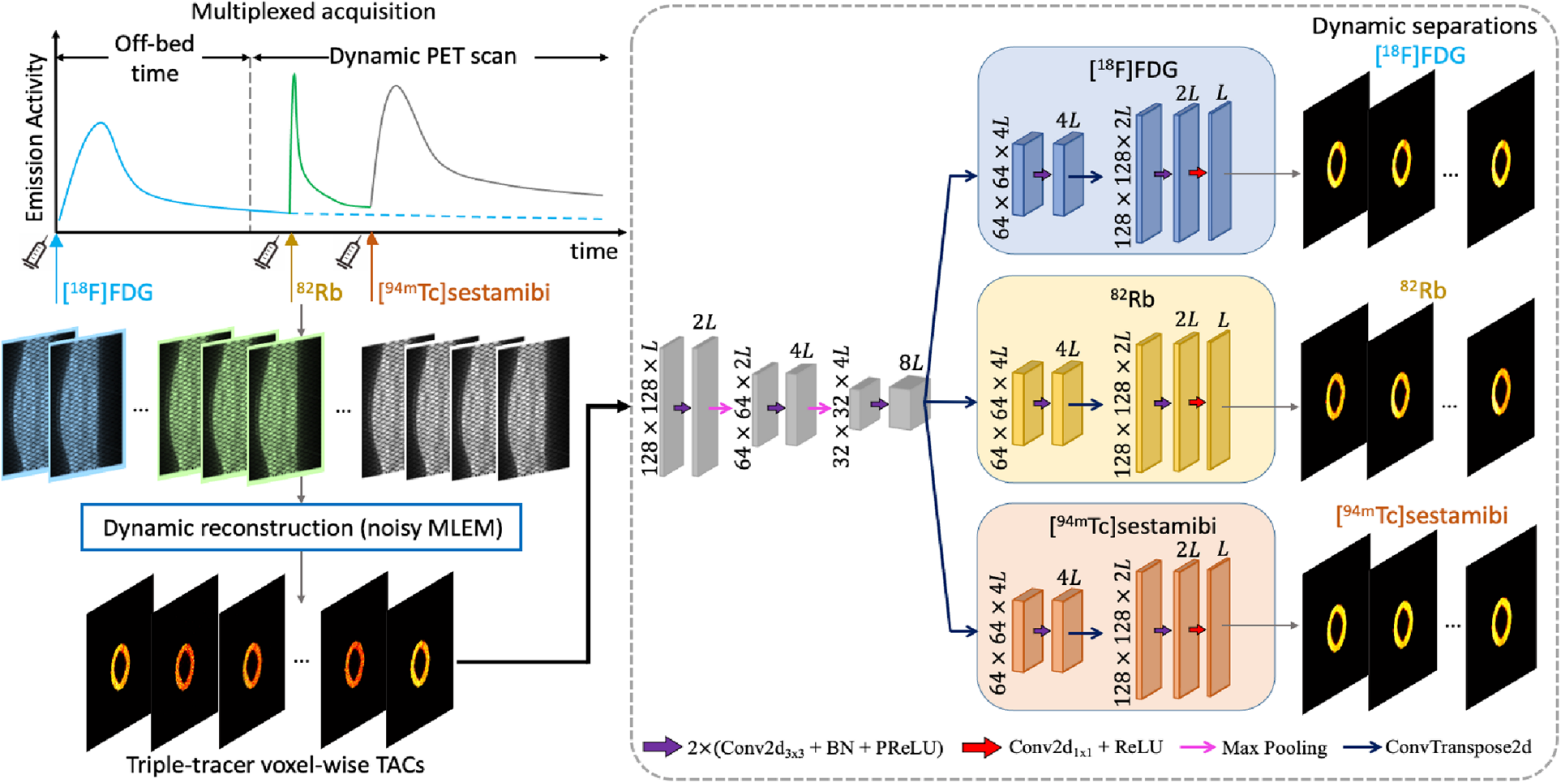
The proposed CED 2D structure. The input is the triple-tracer activity images (voxel-wise TACs with L time frames). The information shown in the plot is based on the simulation study described in Section 3.

The overview of the proposed deep learned post-separation framework for triple-tracer PET image separation is illustrated in Figure 1. In our proposed DL-based method, the triple-tracer activity images (voxel-wise TACs) were used as the network input and the single-tracer activity images were used as the training labels.

We employed an architecture based on the CED, which has been widely used in the DL-based mPET image separation (31, 33, 38–40). In the proposed network, the encoder branches consist of the repeated application of two 3×3 2D convolution layers, each followed by a batch normalisation (BN) and a parametric rectified linear unit (PReLU), in addition to a max-pooling layer for downsampling, followed by the BN and the PReLU. Each of the decoder branches consists of a 3×3 2D transposed convolution layer for upsampling and two 3×3 2D convolution layers, each followed by the BN and the PReLU, and a 1×1 2D convolution layer at the end of each decoder branch. In addition, we activated the output layer using a ReLU to enforce the non-negativity constraint on the separated single-tracer activity images. The number of trainable parameters for the proposed CED 2D is approximately $2.1\times 10^{6}$. The mean squared error (MSE) loss is applied to the activity images of each tracer, and their sum $L_{\text{total}}$ is used as the loss function for network training, which is given by Equation 4:

(4) $$L_{\text{total}}=\sum_{n=1}^{3}\sum_{s=1}^{S}\frac{1}{S}\|{\overset{\circ }{C}}_{s}^{\left(n\right)}-{\hat{C}}_{s}^{\left(n\right)}{\|}_{2}^{2},$$

where ${\overset{\circ }{C}}^{\left(n\right)}$ denotes the activity images of the nth tracer in the network output, ${\hat{C}}^{\left(n\right)}$ is the activity images of the label single-tracer, and S is the total number of training pairs.

## 3 Simulation and validation

### 3.1 Data simulation

**Figure 2:**
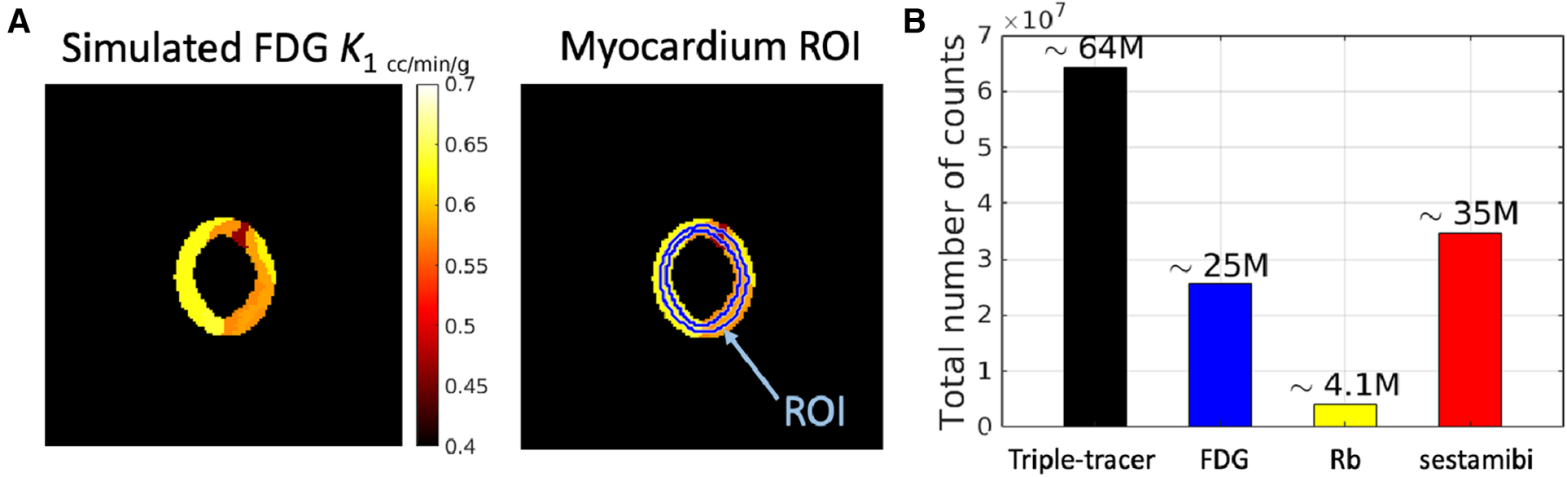
A simulated example of (**A**) ground-truth $K_{1}$ parametric map of FDG (with 7 local regions) along with the pre-defined myocardium ROI (the ring shape indicates the whole myocardium region), and (**B**) total number of counts in a dynamic triple-tracer scan and dynamic single-tracer scans.

We have focused on the simulation study of the triple-tracer myocardium PET image separation based on the combination of [F18]FDG, Rb82, and [Tc99m]sestamibi with decay constants λ[F18]=log⁡(2)/109.7min−1, λ[Rb82]=log⁡(2)/1.26min−1 and λ[Tc99m]=log⁡(2)/52min−1. A simulation was performed to assess the performance of the proposed DL-based triple-tracer separation method. A myocardium dataset was obtained from the 3D MRI cardiac scans provided in the M&Ms challenge^2^ (43). Myocardium segments were extracted from two non-continuous slices (short-axis view), which were selected from one of the frames of each 3D dynamic MRI image. A total of 140 myocardium segment images were obtained. Each myocardium segment image was resized to 128×128 with a voxel size of 2.602×2.602 ${\mathrm{mm}}^{2}$, and further randomly divided into 4 to 14 sub-regions with well-defined boundaries. The ground-truth kinetic parameter for a given sub-region was sampled from a Gaussian distribution with mean values derived from the literature (44–46) (see Table 1)^3^ and coefficient of variation equal to 0.1 to simulate heterogeneous variation within the whole myocardium region (absolute values were taken after sampling). A simulated ground-truth $K_{1}$ parametric map of FDG is shown in Figure 2A (for plotting only, we restricted the voxel values to [0.4, 0.7] to demonstrate the sub-region segments), along with the pre-defined myocardium ROI. The AIFs of each tracer were generated along the shape of the AIFs from the literature (44–46) using Feng’s input function model (47). To further simulate the population variation in the myocardium dataset, the parameters of Feng's input function model were also modelled as a Gaussian variable with a coefficient of variation equal to 0.1 (39), and the absolute values were taken after sampling.

**Table 1:** Mean values of ground-truth kinetic parameters of the myocardium tissue.

	$K_{1}$	$k_{2}$	$k_{3}$	$k_{4}$	$V_{B}$
FDG	0.6	1.2	0.1	–	0.38
Rb	1.4822	0.3159	0.004	–	0.38
sestamibi	0.4	0.094	0.02	0.007	0.38

Units: $K_{1}$: cc/min/g; $k_{2}$–$k_{4}$: ${\text{min}}^{-1}$; $V_{B}$: unit-less.

**Figure 3:**
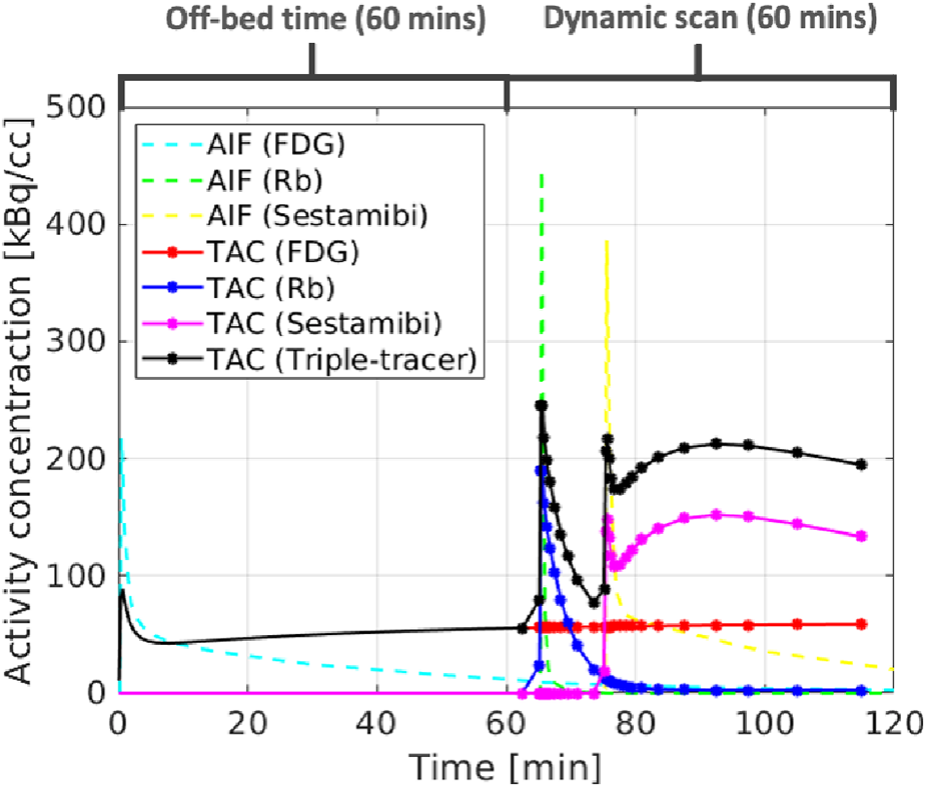
The simulated AIFs (dashed lines), single-tracer TACs (red, blue and magenta) and triple-tracer TACs (black).

The ground-truth single-tracer voxel-wise TACs were generated from the simulated parametric maps using the irreversible two-tissue compartment model for FDG and Rb, and the reversible two-tissue compartment model for sestamibi. The single-tracer voxel-wise TACs were then summed up together to form the ground-truth triple-tracer voxel-wise TACs. In this study, we propose a realistic protocol for the dynamic triple-tracer PET scan, which was conducted for 60 min after the [F18]FDG injection (with an initial 60 min off-bed time). Rb82 was injected 5 min after the start of the dynamic PET scan, followed by the injection of [Tc99m]sestamibi with a 10 min delay. An example of the simulated AIFs and the ROI TACs without decay correction based on the proposed scan protocol is shown in Figure 3.

For the reconstruction of the simulated data, we modelled a GE Discovery ST PET-CT scanner with a system sensitivity of ∼2 cps/kBq in 2D mode (48). Noise-free sinogram data were generated by forward-projecting the dynamic ground-truth images (for both triple-tracer and single-tracer) using a pre-calculated system matrix. A 20% uniform background was included as a simple model for the mean of the random and scatter background events. A scaling factor was applied to this projected data in order to generate the mean count levels in the sinogram, prior to the introduction of Poisson noise into each sinogram bin. This scaling factor was chosen to obtain datasets containing mean total counts in each 2D sinogram, where the mean total counts were modelled based on the system sensitivity of the scanner (Figure 2B).

The dynamic PET scan (60 min for triple-tracer and single-tracer) was divided into 28 time frames: 1×5 min, 4×0.25 min, 2×0.5 min, 3×1 min, 1×2 min, 1×3 min, 4×0.25 min, 2×0.5 min, 3×1 min, 1×2 min, 1×3 min, 3×5 min, 2×10 min. Dynamic images were reconstructed using the MLEM algorithm (initialised by uniform images) with 128 iterations without post-smoothing. The reconstructed images were frame-length corrected and thus equivalent to reconstructed tracer activity images (voxel-wise TACs).

### 3.2 Implementation details and reference methods

For the proposed DL-based method, the dynamic triple-tracer noisy MLEM reconstruction was used as the network input, and the dynamic single-tracer noisy MLEM reconstructions were used as the training labels. We compared the separation performance of the triple-tracer (TT) with the dual-tracer (DT) and the single-tracer (ST) using the proposed DL-based method. For the dual-tracer separation, dynamic dual-tracer ([F18]FDG and Rb82) noisy MLEM reconstructions were used as the network input and the loss function $L_{\text{total}}$ (with n=2) was applied to [F18]FDG and Rb82 only, and dynamic noisy single-tracer MLEM reconstructions were used as training labels. For the single-tracer case, the dynamic noisy single-tracer MLEM reconstruction was used as the network input. To have a fair comparison, the dynamic noisy single-tracer MLEM reconstructions were also used as training labels (with n=1 in $L_{\text{total}}$). All network training was performed in the same manner. In total, 120 simulated data examples were used for training, 10 for validation and 10 for testing. The network parameters were initialised using the Xavier initialisation. The Adam algorithm (49) was used with a learning rate of $5\times 10^{-4}$ and a batch size equal to 8 for network training. To prevent overfitting, all networks were trained for 1,500 epochs with early stropping if there was no improvement in the validation metrics. The network training and evaluation steps were implemented in PyTorch, on a PC with an NVIDIA GeForce RTX 3,090 GPU.

We also compared the DL-based method with the voxel-wise MTCM-based method (v-MTCM) (14, 16). The v-MTCM method estimates the single-tracer TACs by fitting the triple-tracer kinetic model as shown in Equation 1 (with known AIFs of each tracer^4^) to the measured triple-tracer TACs, i.e., the dynamic triple-tracer noisy MLEM, using voxel-wise weighted least squares (VWLS). Note that the fitting was only performed on the frames starting at 60 min after FDG injection, i.e., on the dynamic triple-tracer images obtained within the 60 min triple-tracer PET scan. The time frame durations were used as weighting factors to compensate for non-uniform temporal sampling (16). The trust-region-reflective algorithm was used to perform the VWLS fitting. The stopping criteria were set such that the optimisation procedure was stopped when the relative termination tolerance of the objective function was less than $1\times 10^{-8}$ or the maximum number of iterations (1,600 iterates) was achieved (21). The initial values of the kinetic parameters κ were set to be 0.01 for all voxels and the values of the lower bounds for each parameter were set to be $1\times 10^{-5}$, while the values of the upper bounds for $V_{B}$, $K_{1}$ and $k_{2}-k_{4}$ were set to be [1,5,2,1,1,1], respectively.

Both the DL-based and the MTCM-based methods can be implemented at the ROI level for triple-tracer ROI-TAC separation. The triple-tracer ROI TACs were extracted from the dynamic triple-tracer noisy MLEM followed by a 1D TAC separation. For the DL-based method, we simply replaced the 2D modules in the proposed network shown in Figure 1 with their 1D versions (CED 1D). The number of trainable parameters for the CED 1D is approximately $7.7\times 10^{5}$. The triple-tracer ROI TACs were used as the network input and the single-tracer ROI TACs extracted from the dynamic single-tracer noisy MLEM were used as the training labels. To perform the MTCM-based method at the ROI level (ROI-MTCM), the triple-tracer compartment model was fitted to the extracted triple-tracer ROI TACs to recover the ROI TACs of each tracer using the single-voxel WLS. Both the CED 1D and the ROI-MTCM methods were implemented in the same manner as described for the voxel-level separation.

### 3.3 Evaluation metrics

#### 3.3.1 Voxel-level bias-variance analysis

The separation performance of the different methods was evaluated over R=20 different noise realisations using the voxel-level normalised root mean square error (NRMSE) (Equation 5)

(5) $$\mathrm{NRMSE}=\sqrt{{\mathrm{Bias}}^{2}+{\mathrm{SD}}^{2}},$$

with the bias and standard deviation (SD) given by Equation 6:

(6) $$\begin{array}{r} \mathrm{Bias}=\sqrt{\frac{\sum_{\;j\in \Omega }({\bar{x}}_{j}-x_{j}^{\mathrm{Ref}}{)}^{2}}{\sum_{\;j\in \Omega }(x_{j}^{\mathrm{Ref}}{)}^{2}}}\times 100\%,\;\;\\ \mathrm{SD}=\sqrt{\frac{1}{R}\frac{\sum_{r=1}^{R}\sum_{\;j\in \Omega }({\bar{x}}_{j}-x_{j}^{r})}{\sum_{\;j\in \Omega }(x_{j}^{\mathrm{Ref}}{)}^{2}}}\times 100\%, \end{array}$$

where Ω is the total myocardium region, ${\bar{x}}_{j}=\frac{1}{R}\sum_{r=1}^{R}x_{j}^{r}$ is the mean value for voxel j in the separated image x, obtained by taking the average of the R noise realisations, and $x^{\mathrm{Ref}}$ is a reference image for the error calculation. The single-tracer noise-free (NF) MLEM reconstructions (initialised by uniform images, with 128 iterations) were used as the reference image in all cases.

#### 3.3.2 ROI-level bias-variance analysis

The separated single-tracer TACs were extracted from a pre-defined ROI, as shown on the right-hand side in Figure 2A. The TAC-NRMSE values were also calculated to evaluate the ROI-TAC quantification (Equation 7)

(7) $${\mathrm{NRMSE}}_{\mathrm{TAC}}=\sqrt{{{\mathrm{Bias}}_{\mathrm{TAC}}}^{2}+{{\mathrm{SD}}_{\mathrm{TAC}}}^{2}},$$

with the TAC-bias and TAC-SD given by Equation 8:

(8) $$\begin{array}{r} {\mathrm{Bias}}_{\mathrm{TAC}}=\frac{\left|\bar{c}-c^{\mathrm{Ref}}\right|}{c^{\mathrm{Ref}}}\times 100\%,\;\\ {\mathrm{SD}}_{\mathrm{TAC}}=\frac{1}{c^{\mathrm{Ref}}}\sqrt{\frac{1}{R}\sum_{r=1}^{R}(c^{r}-\bar{c}{)}^{2}}\times 100\%, \end{array}$$

where $c^{\mathrm{Ref}}$ is the single-tracer ROI TACs extracted from the dynamic single-tracer noise-free MLEM, and $\bar{c}=\frac{1}{R}\sum_{r=1}^{R}c^{r}$ denotes the mean of the R noise realisations, and $c^{r}$ is the ROI TACs with the mean ROI uptake in each time frame in the rth realisation.

## 4 Results

### 4.1 Separated image quality

**Figure 4:**
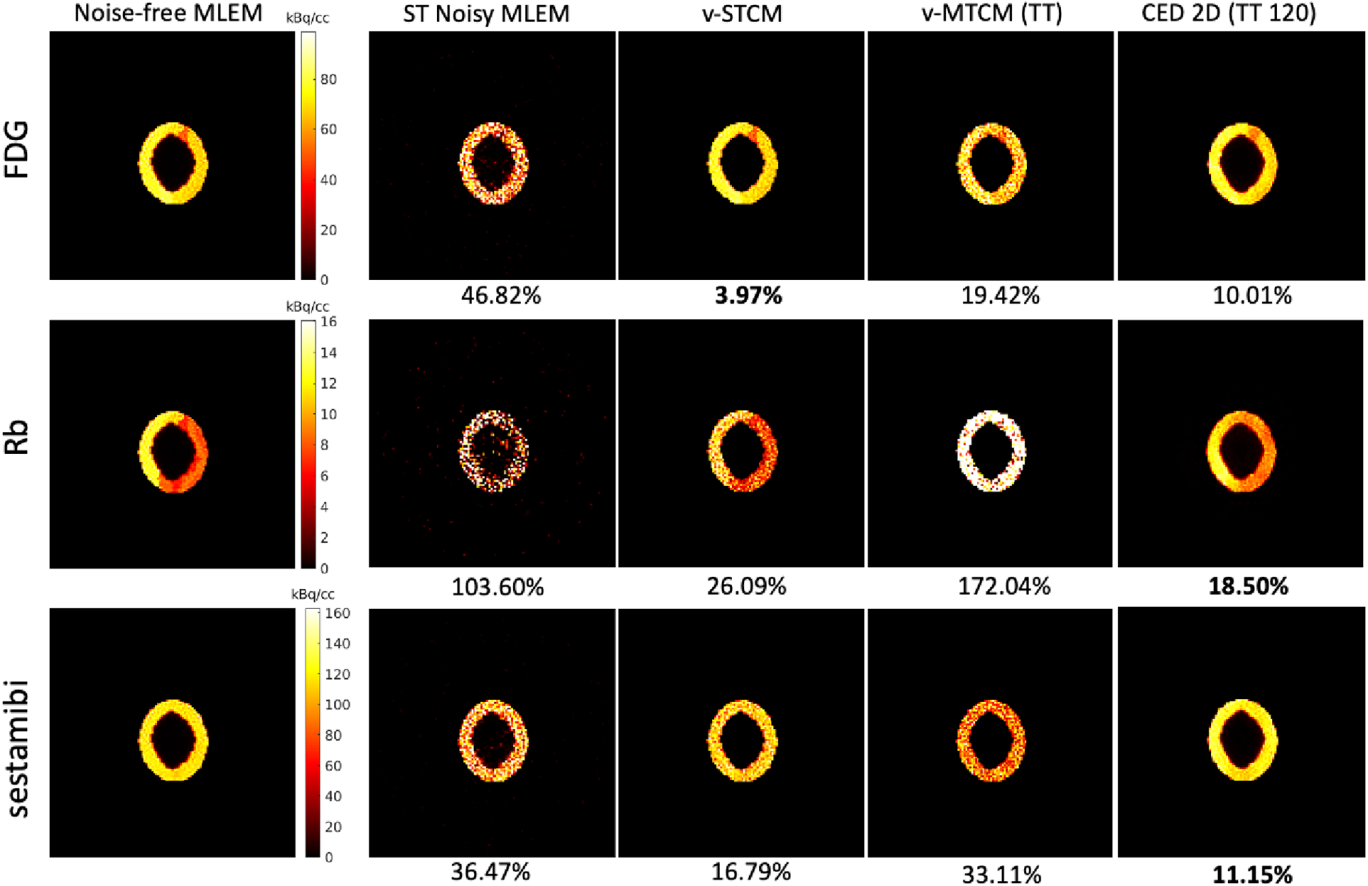
A test example of the single-tracer noise-free and noisy MLEM activity images and the separated activity images of each tracer by different methods for frame 16. The NRMSE values of each separated image are shown at the bottom.

**Figure 5:**
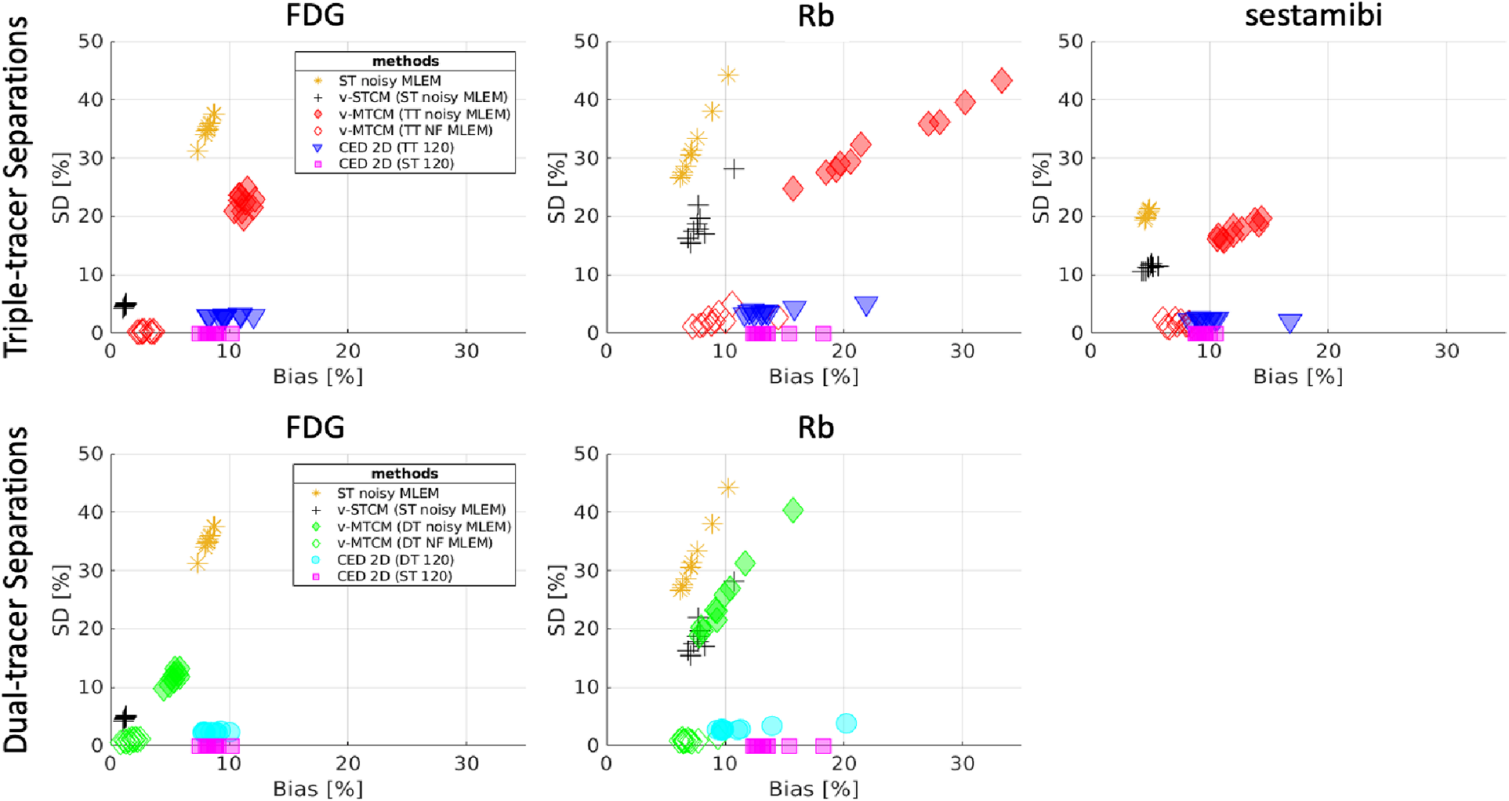
Bias and SD trade-off (over 10 test data) for the separated single-tracer activity images (all time frames were considered) by using different methods. Top-row: performance for triple-tracer separations, bottom-row: performance for dual-tracer separations.

Figure 4 shows the single-tracer noise-free MLEM and the separated single-tracer activity images by different separation methods for frame 16 (a 15-s frame, at 1 min after the injection of [Tc99m]sestamibi). It can be observed that the triple-tracer separation using the CED 2D (with 120 training examples) can substantially reduce the image noise compared to the triple-tracer v-MTCM separation. Figure 5 shows the quantification results of the separated single-tracer activity images (all time frames were considered). The performance of the triple-tracer separations by using different methods is shown in the top row. For the single-tracer activity images, lower bias and SD were found by fitting the compartment model to the single-tracer noisy MLEM voxel by voxel (v-STCM) for FDG, Rb and sestamibi (moving from the yellow cluster to the black cluster), which is consistent with the visual impression of the activity images shown in Figure 4 (by comparing the second and third columns). Although the single-tracer CED 2D further reduces the SD compared to the v-STCM, a higher bias was still obtained due to the small number of training pairs and the prior of the proposed network.

For the considered triple-tracer separation, the situation worsens. High bias was obtained for each tracer using the v-MTCM method for the noisy triple-tracer separation compared to the single-tracer cases. The main reason is that the MTCM-based method is sensitive to noise and may fall into local minima even when the noise level is low (21). This can be verified by comparing the performance of the v-MTCM method for noise-free triple-tracer separation,^5^ where the SD is much lower, but the bias level is still higher than the single-tracer noisy v-MTCM. The DL-based method, CED 2D, sufficiently reduces the SD and achieved lower bias compared to the v-MTCM method for noisy triple-tracer separation even though the network was trained using noisy labels. The CED 2D uses both spatial and temporal information for triple-tracer separation, while the v-MTCM method only uses temporal information and is highly dependent on prior information, such as the time delay intervals between tracer injections (33, 42). More importantly, the CED 2D with MSE loss learns to output the mean of all plausible noisy explanations when it is trained using noisy labels, and thus the proposed network implicitly learns to denoise the output images (33). However, the CED 2D for triple-tracer separation fails to reach the same level of bias and SD as the CED 2D for single-tracer using the same number of training examples, indicating that the triple-tracer separation task is still challenging for the proposed DL-based method.

Dual-tracer ([F18]FDG and Rb82) separation was also investigated. The bias and SD trade-off is shown in the bottom row of Figure 5. The CED 2D results in lower SD but higher bias compared to the v-MTCM for noisy dual-tracer separation for both FDG and Rb. The lower bias level in the v-MTCM separation is due to several factors: (i) the very short half-life of Rb82 (76-s), (ii) the injection of Rb82 was delayed by 5 min after the start of the dynamic PET scan, i.e., only the [F18]FDG signal was measured in the first 5 min, and (iii) the activity concentration of [F18]FDG tends to become stable (see the rather flat red line in Figure 3). These factors also make the dual-tracer signals become much easier to disentangle compared to the triple-tracer separation task, leading to lower bias and SD in the dual-tracer separation compared to the triple-tracer case for both the v-MTCM and the CED 2D. We also noted that the bias and SD of Rb are higher than those of FDG and sestamibi for the single-tracer, the dual-tracer, and the triple-tracer cases. This is because the MLEM reconstructions of Rb were extremely noisy due to the low-count level caused by the short half-life of Rb82 and the short time frame duration in the early time frames.

### 4.2 Impact of the number of training examples

**Figure 6:**
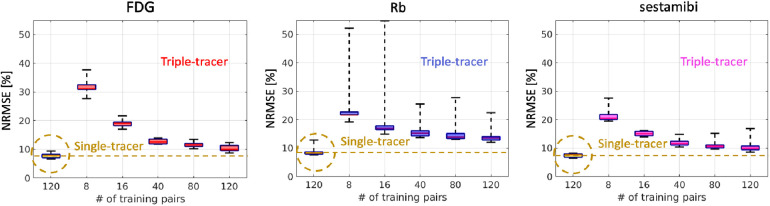
NRMSE levels (over 10 test data) of the separated single-tracer activity images (all time frames were considered) by using the triple-tracer CED 2D with different numbers of training examples. The yellow dashed lines indicate the median of the NRMSE of each tracer using the single-tracer CED 2D.

We retrained each CED 2D using different sample sizes to assess the impact of the number of training examples on triple-tracer separation. Figure 6 shows that the NRMSE values of the separated single-tracer activity images decrease as the increasing number of training examples (from 8 to 120). With 120 training examples, the CED 2D triple-tracer separation achieved ∼12%, ∼15% and ∼12% NRMSE values for FDG, Rb and sestamibi, respectively. However, these errors are still higher than those of the CED 2D for single-tracer using the same number of training examples (120 pairs), which again shows the bottleneck of the proposed DL-based separation method.

### 4.3 Parametric map separation

**Figure 7:**
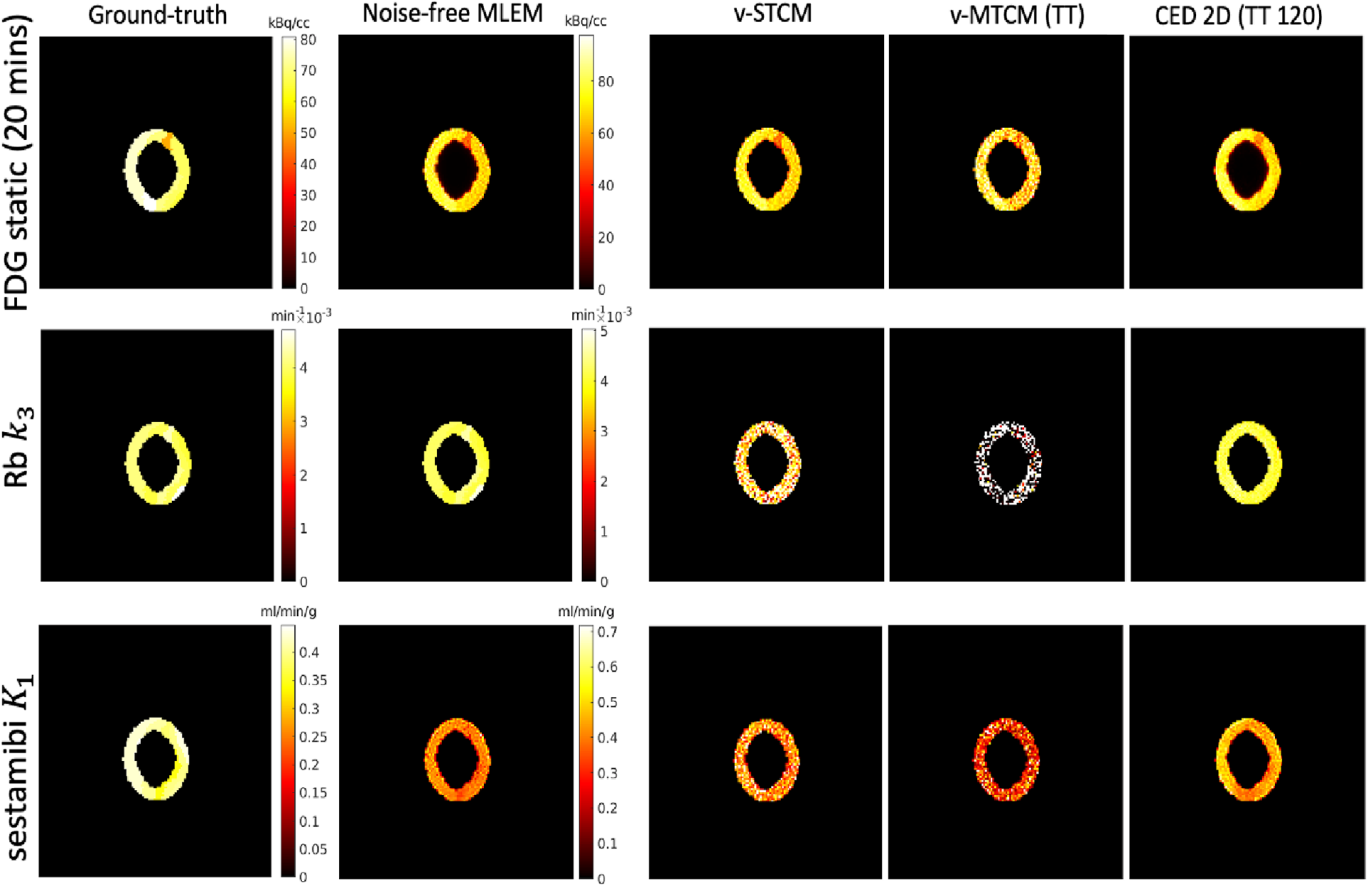
A test example of the single-tracer ground-truth, the single-tracer noise-free MLEM parametric maps and the separated parametric maps of each tracer by different methods.

Parametric imaging was also performed on the separated activity images. The v-STCM method was used to estimate the parametric maps from the separated single-tracer images obtained from the CED 2D. Note that in the v-MTCM method, the parametric images of each tracer were separated before recovering the single-tracer activity images, eliminating the need for post-estimation. The parametric maps recovered from the single-tracer noise-free MLEM^6^ were used as reference images. Figure 7 shows the parametric images of [F18]FDG for a static image corresponding to the last 20-min interval obtained by frame integration, Rb82 delivery rate $k_{3}$, and [Tc99m]sestamibi delivery rate $K_{1}$. Compared with the parametric images estimated from the triple-tracer v-MTCM separated activity images, the CED 2D results are more similar to the reference images.

**Figure 8:**
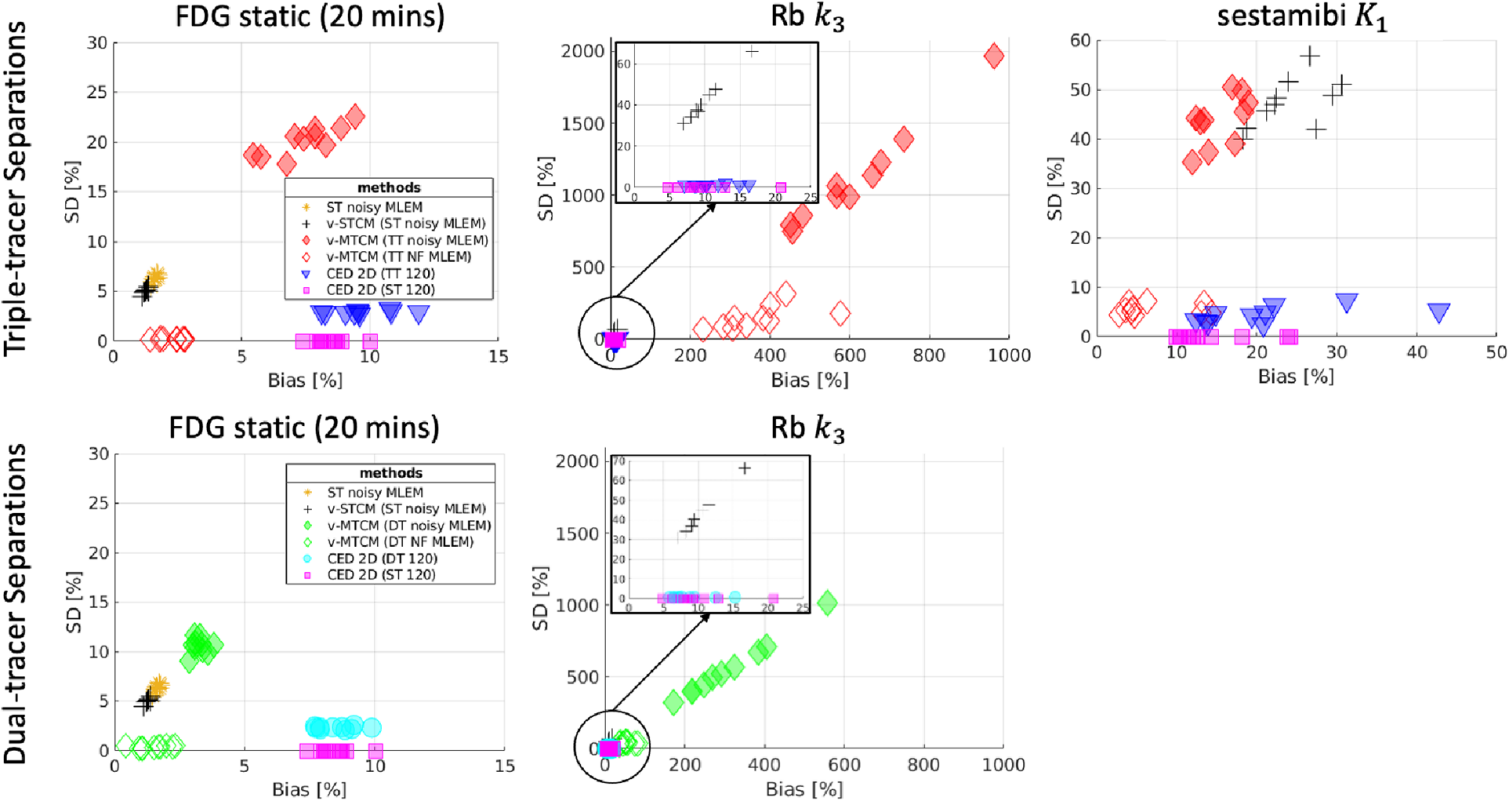
Bias and SD trade-off (over 10 test data) for the separated parametric maps. Top-row: performance of triple-tracer separations, bottom-row: performance of dual-tracer separations.

Figure 8 shows the corresponding bias and SD trade-off of the separated parametric images. Without the impact of noise, the parametric maps obtained from the noise-free v-MTCM separation achieved much lower bias and SD compared to the noisy v-MTCM separation for both the triple and dual-tracer separations. The parametric maps obtained from the CED 2D separation exhibit a dramatic reduction in SD compared to the noisy v-MTCM separation. In addition, lower bias and SD of the parametric maps were achieved in the dual-tracer separations compared to the triple-tracer separations for both the v-MTCM and the CED 2D (see Subsection 4.1 for detailed discussion). However, the bias of the static FDG and sestamibi $K_{1}$ estimated from the triple-tracer and dual-tracer separations using the v-MTCM method is lower than that of the CED 2D, indicating the lack of training examples and the weak inductive prior of the CED 2D. The Rb $k_{3}$ images generally have higher bias and SD compared to the FDG static and sestamibi $K_{1}$ images using the v-MTCM, demonstrating that the separation and estimation of Rb $k_{3}$ is still challenging. However, a clear reduction in bias and SD for $k_{3}$ was achieved by using the CED 2D, which is consistent with the observations in Figure 7.

### 4.4 ROI-TAC separation via CED 1D

**Figure 9:**
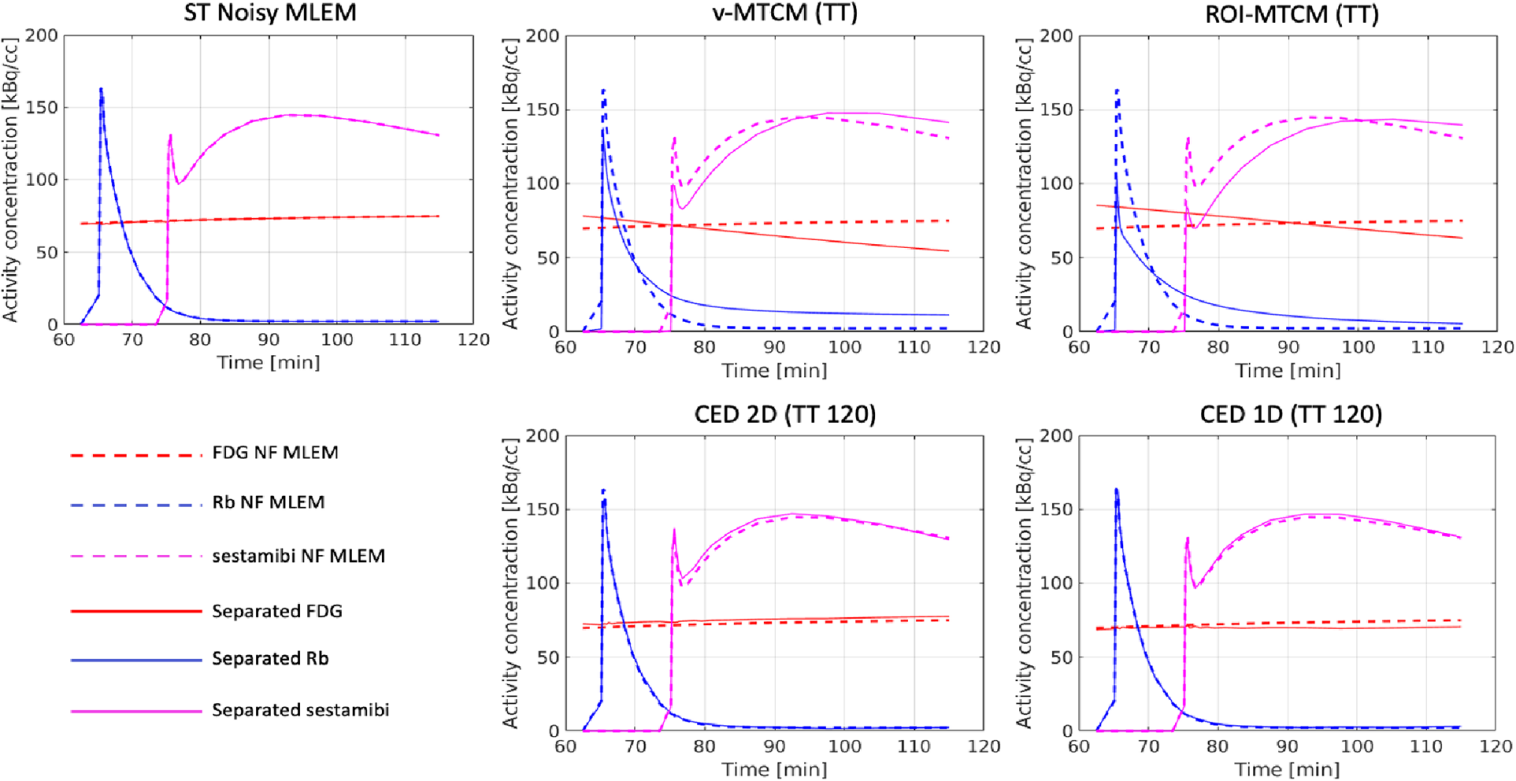
A test example of the mean separated (non-decay corrected) TACs in the pre-defined myocardium ROI over 20 noise realisations.

The separated ROI TACs using the MTCM-based and DL-based methods were also assessed. Figure 9 shows the mean separation results of a single test example over 20 different noise realisations. The reference single-tracer ROI TACs extracted from the single-tracer noise-free MLEM (dashed lines), the separated ROI TACs using the voxel-level methods (v-MTCM and CED 2D) and the ROI-level methods (ROI-MTCM and CED 1D) are presented. Both the CED 2D and the CED 1D generally resemble the reference TACs of each tracer compared to the v-MTCM and the ROI-MTCM.

**Figure 10:**
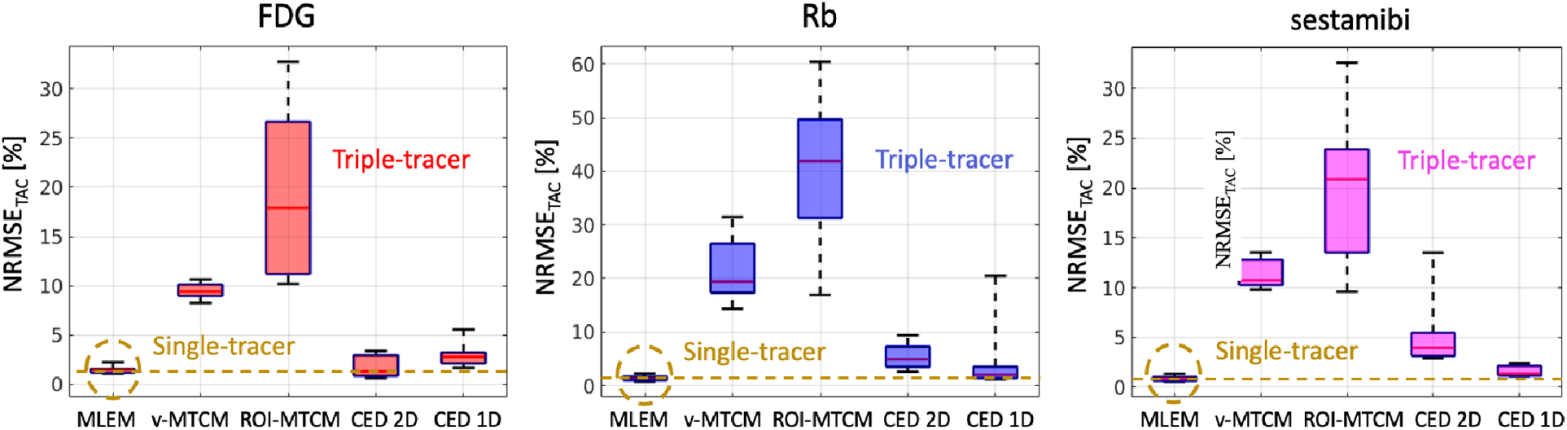
NRMSE (over 10 test data) of the separated ROI TACs (non-decay corrected) using the triple-tracer v-MTCM and CED at the voxel (2D) and ROI (1D) level. The yellow dashed lines indicate the median of the NRMSE of the ROI TACs extracted from the single-tracer noisy MLEM.

The NRMSE of the separated TACs are shown in Figure 10. For the MTCM-based separation methods, the v-MTCM results in much lower NRMSE for all tracers compared to the ROI-MTCM, indicating that the separation performance was enhanced for the MTCM-based method by considering the voxel-wise separation in the myocardium ROI. On average, a significant reduction in NRMSE was achieved by the CED 2D and the CED 1D compared to the v-MTCM and the ROI-MTCM for all tracers. The NRMSE of the CED 1D is higher than that of the CED 2D for FDG, while it is lower for Rb and sestamibi. We observed that the ROI TAC of FDG became stable (see the rather flat red dashed lines in Figure 9). The CED 2D implicitly learned to denoise the output images, which is beneficial for the separation of the rather flat FDG ROI TAC. However, the Rb and sestamibi ROI TACs each contain a peak at the very beginning, which is more difficult to recover in the separation compared to the FDG ROI TAC. In this case, the CED 1D results in a better separation performance. The CED 1D learns a direct mapping to separate single-tracer TACs from triple-tracer TACs in the time domain, which is more effective, whereas the CED 2D is an indirect method where single-tracer TACs are extracted from single-tracer images after the separation in the image domain. However, the NRMSE of the separated TACs using either CED 1D or CED 2D in general fails to reach the same level as the single-tracer noisy MLEM as highlighted by the dashed yellow lines.

## 5 Discussion

In this paper, we demonstrated the feasibility of deep learned triple-tracer ([F18]FDG, Rb82 and [Tc94m]sestamibi) myocardium PET image separation using simulated data. We simulated the myocardium phantoms based on slices extracted from real patient cardiac MRI images. The ground-truth activity images of each tracer were generated from the simulated parametric maps with the AIFs generated based on Feng’s input function model.

The conventional MTCM methods and the proposed DL-based method were investigated in the simulation study. The v-MTCM method only uses temporal information for the separation, and the fitting process was sensitive to noise and may suffer from local minima, leading to the poor quality of the separated images. The proposed DL-based separation (CED 2D) can dramatically improve the separated image quality by using both spatial and temporal information for triple-tracer separation. Considering only the dual-tracer ([^18^F]FDG and ^82^Rb), the v-MTCM method achieves a lower bias compared to the CED 2D for the separated activity images. This is because, in the dual-tracer case, the very short half-life of Rb82, the delayed injection time of Rb82 and the almost constant activity concentration of [F18]FDG as background offers strong prior information for the v-MTCM separation. However, the performance of the v-MTCM method still suffers from noise, resulting in a higher SD compared to the CED 2D separation. The separation of the parametric maps was also investigated. The CED 2D significantly reduces the noise in the separated parametric images compared to the v-MTCM method. Compared to the CED 2D, lower bias was obtained for FDG static and sestamibi $K_{1}$ using the v-MTCM for both triple and dual-tracer separations, indicating that the proposed DL-based method still suffers from the lack of training pairs and the weak inductive prior of the network. The separation of the triple-tracer ROI TACs was also evaluated, showing that the v-MTCM separation results in lower NRMSE values compared to those of the ROI-MTCM separation. The CED 2D and the CED 1D can further reduce the NRMSE markedly. In addition, the CED 1D offers a better ROI-TAC separation compared to the CED 2D for Rb82 and [Tc99m]sestamibi, where their ROI-TACs contained a peak at the beginning. When the ROI TACs are rather flat, as shown for [F18]FDG, the CED 2D gives a better separation performance compared to the CED 1D. The results of the simulation study show a promising direction and also provide guidance for the DL-based separation method in future physical phantom experiments and real patient data studies.

This study has several limitations, five of which are discussed below. (1) The study only considered the myocardium tissue, overlooking the fact that the blood pool region is also imaged in dynamic cardiac PET scans. The AIFs could therefore potentially be extracted from the blood-pool region in the dynamic PET images and integrated into the proposed DL-based method as prior information to facilitate the mPET separation. (2) The current study exclusively focuses on the triple-tracer separation of myocardium PET images using the tracer combination of [F18]FDG, Rb82 and [Tc99m]sestamibi. Additional investigations are required to explore the application of the proposed framework to other mPET separation tasks, such as the separation of mPET brain images using different tracer combinations. (3) The impact of (i) relative and absolute injection dose on the triple-tracer imaging, (ii) the order of tracer injection, and (iii) the scanning protocol, were not investigated in this study. (4) The present work only focuses on the separation for 2D PET images whereas conventional PET imaging is typically conducted in 3D. Even when 3D PET imaging is considered, the low-count levels encountered in short time frames can lead to extremely noisy MLEM reconstructions, which makes the task of mPET image separation even more challenging. With new techniques to improve the quality of reconstructed PET images (50) and the arrival of new scanners, e.g., total body PET, the higher-quality image data could be utilised to further improve the mPET separation. (5) The present study is based on simulated data. The proposed model would need to be investigated and validated rigorously on physical phantoms or synthetic data to assess its feasibility for real data applications.

The proposed pure data-driven approach usually uses over-parameterised networks with only a very weak inductive prior and requires a large amount of training data, while the availability of mPET data is typically limited. To acquire data for network training (in a supervised manner), in real practice, each patient would need to undergo three independent single-tracer dynamic PET scans (used as training labels) and one triple-tracer dynamic PET scan (used as network input), resulting in a long scan duration for a given patient. Additionally, compared to dynamic PET imaging, short-duration static imaging is by far the more commonly encountered clinical imaging protocol for diagnosis and treatment monitoring. Therefore, acquiring a large number of training datasets is challenging in practice. A potential future direction to improve DL-based mPET separation with less training data is to incorporate a stronger inductive prior, such as kinetic modeling, into the deep network (51).

Although the current study has shown the robustness of using DL for mPET separation, the ability to generalise to other tracer combinations or images that may lie outside the training distribution still remains a concern. A future study will investigate the use of fine-tuning of a pre-trained network (trained using phantom and Monte Carlo simulation data) with unseen data or real patient data in a self-supervised manner (52) to improve its generalisation ability.

## 6 Conclusions

We have developed a DL-based method for triple-tracer myocardium PET image separation and demonstrated the results of the proof-of-concept study based on simulated data. Unlike from the conventional MTCM method, the proposed DL-based method separates the triple-tracer PET image without explicitly knowing the AIFs of each tracer. As compared to the MTCM separation, the proposed method uses spatiotemporal information for the separation, which improves the separation performance at both the voxel and ROI level. The simulation study also demonstrates the feasibility and potential of the proposed DL-based method for the application in pre-clinical and clinical studies.

## Data Availability

The raw data supporting the conclusions of this article will be made available by the authors, without undue reservation.

## Footnotes

^1^Note that the decay correction cannot be performed in the multi-tracer TACs before separation because the proportion of each tracer (and thus its decay correction factor) is unknown prior to separation. Although we do model decay in the Equation 1, we do not seek to include decay correction in the present work.

^2^ https://www.ub.edu/mnms/

^3^The kinetic parameters used for the [Tc94m]sestamibi TAC simulations are the same as those used in the literature (44), but for the isotope Tc99m.

^4^In the simulation study, we focused on the validation of the algorithm and thus the AIFs are assumed to be known.

^5^The realisations of noise-free triple-tracer separation using the v-MTCM method were obtained by performing re-runs of the VWLS using different random initialisations of the kinetic parameters κ.

^6^Note that the noise-free MLEM parametric images are still far away from the ground-truth because of the early termination of the MLEM algorithm (128 iterations).

## References

[1] Gunn RN Slifstein M Searle GE Price JC. Quantitative imaging of protein targets in the human brain with PET. Phys Med Biol. (2015) 60:R363. 10.1088/0031-9155/60/22/R363 26513176

[2] Machac J. Cardiac positron emission tomography imaging. Semin Nucl Med. (2005) 35:17–36. 10.1053/j.semnuclmed.2004.09.002 15645392

[3] Pantel AR Mankoff DA Karp JS. Total-body PET: will it change science, practice? J Nucl Med. (2022) 63:646–8. 10.2967/jnumed.121.263481 35273091 PMC12079751

[4] Bengel FM Higuchi T Javadi MS Lautamäki R. Cardiac positron emission tomography. J Am Coll Cardiol. (2009) 54:1–5. 10.1016/j.jacc.2009.02.065 19555834

[5] Karakatsanis NA Lodge MA Zhou Y Mhlanga J Chaudhry MA Tahari AK Dynamic multi-bed FDG PET imaging: feasibility, optimization. In: IEEE Nuclear Science Symposium Conference Record (2011). p. 3863–70.

[6] Sciagrà R Lubberink M Hyafil F Saraste A Slart RHJA Agostini D EANM procedural guidelines for PET/CT quantitative myocardial perfusion imaging. Eur J Nucl Med Mol Imaging. (2021) 49:1040–69. 10.1007/s00259-020-05046-9 PMC7603916 33135093

[7] Matsuo S Nakajima K Kinuya S. Evaluation of cardiac mitochondrial function by a nuclear imaging technique using technetium-99m-MIBI uptake kinetics. Asia Ocean J Nucl Med Biol. (2013) 1:39. 10.7508/aojnmb.2013.01.008 27408841 PMC4937671

[8] Kadrmas DJ Hoffman JM. Methodology for quantitative rapid multi-tracer PET tumor characterizations. Theranostics. (2013) 3:757. 10.7150/thno.5201 24312149 PMC3840410

[9] Figueiras FP Jiménez X Pareto D Gómez V Llop J Herance R Simultaneous dual-tracer PET imaging of the rat brain, its application in the study of cerebral ischemia. Mol Imaging Biol. (2011) 13:500–10. 10.1007/s11307-010-0370-5 20652756

[10] Huang SC Carson RE Hoffman EJ Kuhl DE Phelps ME. An investigation of a double-tracer technique for positron computerized tomography. J Nucl Med. (1982) 23:816–22. PMID: 6980975

[11] Verhaeghe J D’Asseler Y Staelens S Lemahieu I. Noise properties of simultaneous dual tracer pet imaging. In: IEEE Nuclear Science Symposium Conference Record 5 (2005). p. 2611–4.

[12] Koeppe RA Ficaro EP Raffel DM Minoshima S Kilbourn MR. Temporally overlapping dual-tracer PET studies. Quant Funct Brain Imaging Positron Emiss Tomogr. (1998): 359–66. 10.1016/B978-012161340-2/50056-1

[13] Koeppe RA Raffel DM Snyder SE Ficaro EP Kilbourn MR Kuhl DE. Dual-[c11] tracer single-acquisition positron emission tomography studies. J Cereb Blood Flow Metab. (2001) 21:1480–92. 10.1097/00004647-200112000-00013 11740210

[14] Kadrmas DJ Rust TC. Feasibility of rapid multitracer PET tumor imaging. IEEE Trans Nucl Sci. (2005) 52:1341–7. 10.1109/TNS.2005.858230

[15] Nishizawa S Kuwabara H Ueno M Shimono T Toyoda H Konishi J. Double-injection FDG method to measure cerebral glucose metabolism twice in a single procedure. Ann Nucl Med. (2001) 15:203–7. 10.1007/BF02987832 11545189

[16] Rust TC Kadrmas DJ. Rapid dual-tracer PTSM+ ATSM PET imaging of tumour blood flow and hypoxia: a simulation study. Phys Med Biol. (2005) 51:61. 10.1088/0031-9155/51/1/005 16357431

[17] Black NF McJames S Rust TC Kadrmas DJ. Evaluation of rapid dual-tracer 62Cu-PTSM+62Cu-ATSM PET in dogs with spontaneously occurring tumors. Phys Med Biol. (2007) 53:217. 10.1088/0031-9155/53/1/015 18182698 PMC3746544

[18] Kadrmas DJ Rust TC Hoffman JM. Single-scan dual-tracer FLT+FDG PET tumor characterization. Phys Med Biol. (2013) 58:429. 10.1088/0031-9155/58/3/429 23296314 PMC3553659

[19] Black NF James SM Kadrmas DJ. Rapid multi-tracer PET tumor imaging with F18-FDG and secondary shorter-lived tracers. IEEE Trans Nucl Sci. (2009) 56:2750–8. 10.1109/TNS.2009.2026417 20046800 PMC2799294

[20] Zhang JL Morey AM Kadrmas DJ. Application of separable parameter space techniques to multi-tracer PET compartment modeling. Phys Med Biol. (2016) 61:1238. 10.1088/0031-9155/61/3/1238 26788888 PMC4765365

[21] Cheng X Li Z Liu Z Sung Cheng Huang NN Keller U Ziegler SI Direct parametric image reconstruction in reduced parameter space for rapid multi-tracer PET imaging. IEEE Trans Med Imaging. (2015) 34:1498–512. 10.1109/TMI.2015.2403300 25700443

[22] Verhaeghe J Reader AJ. Simultaneous water activation and glucose metabolic rate imaging with PET. Phys Med Biol. (2013) 58:393. 10.1088/0031-9155/58/3/393 23296197

[23] Fakhri GE Trott CM Sitek A Bonab A Alpert NM. Dual-tracer PET using generalized factor analysis of dynamic sequences. Mol Imaging Biol. (2013) 15:666–74. 10.1007/s11307-013-0631-1 23636489 PMC3812387

[24] Joshi AD Koeppe RA Fessier JA Kilbourn MR. Signal separation and parameter estimation in noninvasive dual-tracer PET scans using reference-region approaches. J Cereb Blood Flow Metab. (2009) 29:1346–57. 10.1038/jcbfm.2009.53 19401708

[25] Bell C Puttick S Rose S Smith J Thomas P Dowson N. Design and utilisation of protocols to characterise dynamic PET uptake of two tracers using basis pursuit. Phys Med Biol. (2017) 62:4897. 10.1088/1361-6560/aa6b44 28375137

[26] Taheri N Crom BL Bouillot C Chérel M Costes N Gouard S Design of a generic method for single dual-tracer pet imaging acquisition in clinical routine. Phys Med Biol. (2023) 68:085016. 10.1088/1361-6560/acc723 36958048

[27] Andreyev A Celler A. Dual-isotope PET using positron-gamma emitters. Phys Med Biol. (2011) 56:4539. 10.1088/0031-9155/56/14/020 21725143

[28] Fukuchi T Okauchi T Shigeta M Yamamoto S Watanabe Y Enomoto S. Positron emission tomography with additional γ-ray detectors for multiple-tracer imaging. Med Phys. (2017) 44:2257–66. 10.1002/mp.12149 28168704

[29] Fukuchi T Shigeta M Haba H Mori D Yokokita T Komori Y Image reconstruction method for dual-isotope positron emission tomography. J Instrum. (2021) 16:01035. 10.1088/1748-0221/16/01/P01035

[30] Pratt EC Lopez-Montes A Volpe A Crowley MJ Carter LM Mittal V Simultaneous quantitative imaging of two PET radiotracers via the detection of positron–electron annihilation and prompt gamma emissions. Nat Biomed Eng. (2023) 7:1028–39. 10.1038/s41551-023-01060-y 37400715 PMC10810307

[31] Lian D Li Y Liu H. Spatiotemporal attention constrained deep learning framework for dual-tracer PET imaging. In: Annual Conference on Medical Image Understanding and Analysis (2022). p. 87–100.

[32] Qing M Wan Y Huang W Xu Y Liu H. Separation of dual-tracer PET signals using a deep stacking network. Phys Res Sect A. (2021) 1013:165681. 10.1016/j.nima.2021.165681

[33] Pan B Marsden PK Reader AJ. Dual-tracer PET image separation by deep learning: a simulation study. Appl Sci. (2023) 13:7. 10.3390/app13074089

[34] Ruan D Liu H. Separation of a mixture of simultaneous dual-tracer PET signals: a data-driven approach. IEEE Trans Nucl Sci. (2017) 64:2588–97. 10.1109/TNS.2017.2736644

[35] Tong J Wang C Liu H. Temporal information-guided dynamic dual-tracer PET signal separation network. Med Phys. (2022) 49:4585–98. 10.1002/mp.15566 35396705

[36] Tong J Chen Y Liu H. Single-scan dual-tracer separation network based on pre-trained GRU. In: International Workshop on Multiscale Multimodal Medical Imaging (2019). p. 43–50.

[37] Xu J Liu H. Deep-learning-based separation of a mixture of dual-tracer single-acquisition PET signals with equal half-lives: a simulation study. IEEE Trans Radiat Plasma Med Sci. (2019) 3:649–59. 10.1109/TRPMS.2019.2897120

[38] Xu J Liu H. Three-dimensional convolutional neural networks for simultaneous dual-tracer PET imaging. Phys Med Biol. (2019) 64:18. 10.1088/1361-6560/ab3103 31292287

[39] Zeng F Fang J Muhashi A Liu H. Direct reconstruction for simultaneous dual-tracer PET imaging based on multi-task learning. EJNMMI Res. (2023) 13:7. 10.1186/s13550-023-00955-w 36719532 PMC9889598

[40] Wang C Fang J Liu H Gong K. Direct reconstruction and separation for triple-tracer PET imaging based on three-dimensional encoder-decoder network. In: Medical Imaging 2023: Physics of Medical Imaging 12463 (2023). p. 585–593.

[41] Ye YWH Liu H. Deep-learning based joint estimation of dual-tracer PET image activity maps and clustering of time activity curves. In: Medical Imaging 2021: Physics of Medical Imaging 11595 (2021). p. 981–9.

[42] Ding W Yu J Zheng C Fu P Huang Q Feng DD Machine learning-based noninvasive quantification of single-imaging session dual-tracer F18-FDG and Ga68-DOTATATE dynamic PET-CT in oncology. IEEE Trans Med Imaging. (2021) 41:347–59. 10.1109/TMI.2021.3112783 34520350

[43] Campello VM Gkontra P Izquierdo C Martin-Isla C Sojoudi A Full PM Multi-centre, multi-vendor and multi-disease cardiac segmentation: the M&Ms challenge. IEEE Trans Med Imaging. (2021) 40:3543–54. 10.1109/TMI.2021.3090082 34138702

[44] Hsu B Chen F-C Wu T-C Huang W-S Hou P-N Chen C-C Quantitation of myocardial blood flow and myocardial flow reserve with mTc99-sestamibi dynamic SPECT/CT to enhance detection of coronary artery disease. Eur J Nucl Med Mol Imaging. (2014) 41:32294–2306. 10.1007/s00259-014-2881-9 25143072

[45] Karakatsanis NA Lodge MA Zhou Y Mhlanga J Chaudhry MA Tahari AK Dynamic multi-bed FDG pet imaging: feasibility and optimization. In: IEEE Nuclear Science Symposium Conference Record (2011). p. 3863–70.

[46] Lu L Ma X ud Din HM Ma J Feng Q Rahmim A Enhancement of dynamic myocardial perfusion pet images based on low-rank plus sparse decomposition. Comput Methods Programs Biomed. (2018) 154:57–69. 10.1016/j.cmpb.2017.10.020 29249347

[47] Feng D Wong K-P Wu C-M Siu W-C. A technique for extracting physiological parameters and the required input function simultaneously from PET image measurements: theory and simulation study. IEEE Trans Inf Technol Biomed. (1997) 1:243–54. 10.1109/4233.681168 11020827

[48] Mawlawi O Podoloff DA Kohlmyer S Williams JJ Stearns CW Culp RF Performance characteristics of a newly developed PET/CT scanner using NEMA standards in 2D and 3D modes. J Nucl Med. (2004) 45:1734–42. PMID: 15471842

[49] Kingma DP Ba J. Adam: a method for stochastic optimization. *arXiv* [Preprint]. *arXiv:1412.6980* (2014).

[50] Reader AJ Pan B. AI for PET image reconstruction. Br J Radiol. (2023) 96:20230292. 10.1259/bjr.20230292 37486607 PMC10546435

[51] Pan B Marsden PK Reader AJ. Kinetic model-informed deep learning for multiplexed PET image separation. In: 2023 IEEE Nuclear Science Symposium, Medical Imaging Conference and International Symposium on Room-Temperature Semiconductor Detectors (NSS MIC RTSD) (2023). p. 1–2.

[52] Reader AJ. Self-supervised and supervised deep learning for PET image reconstruction. (2023). Available online at: https://arxiv.org/abs/2302.13086.

